# Supplementary material for: Acetylsalicylic Acid Effect in Colorectal Cancer Taking into Account the Role of Tobacco, Alcohol and Excess Weight
Source: Int J Environ Res Public Health. 2023 Feb 24;20(5):4104. doi: 10.3390/ijerph20054104 (PMC10001481; doi:10.3390/ijerph20054104)
Supplement: Supplementary file 1 [file ijerph-20-04104-s001.zip › ijerph-2173534-supplementary.pdf]

Supplementary tables

Table S1. Cox regression stratified by excess weight (obesity-overweight) and normal-weight.

	Normal-Weight		Excess Weight	
	**Adjusted HR; 95% CI**	***p*-Value**	**Adjusted HR; 95% CI**	***p*-Value**
Female		Ref. Group		Ref. Group
Male	1.6 (1.2–2.2)	0.002	1.9 (1.7–2.2)	<0.001
Age [50–59)	-	Ref. Group	-	Ref. Group
Age [60–69)	2.3 (1.6–3.4)	<0.001	1.7 (1.5–2.1)	<0.001
Age [70–79)	3.1 (2.0–4.7)	<0.001	2.2 (1.8–2.6)	<0.001
Age [80–89)	2.4 (1.5–3.9)	<0.001	2.2 (1.7–2.6)	<0.001
Age [90–)	0.03 (0.0–0.1)	<0.001	1.0 (0.6–1.9)	0.7
Aspirin use	0.8 (0.5–1.6)	0.7	0.7 (0.6–0.8)	<0.001
Risky drinking	2.1 (1.0–4.3)	0.04	1.5 (1.1–2.0)	0.004
Smoking	1.7 (1.0–2.8)	0.04	1.5 (1.2–1.7)	<0.001

Ref. group = Reference group; HR = Hazard ratio; HR adjusted by gender, age, aspirin, risky drinking and smoking.

Table S2. Cox regression stratified by aspirin and non-aspirin use.

	Non-Aspirin Use		Aspirin Use	
	**Adjusted HR; 95% CI**	***p*-Value**	**Adjusted HR; 95% CI**	***p*-Value**
Female		Ref. Group		Ref. Group
Male	1.8 (1.6–2.0)	<0.001	2.2 (1.4–3.3)	<0.001
Age [50–59)	-	Ref. Group	-	Ref. Group
Age [60–69)	1.8 (1.5–2.1)	<0.001	1.7 (0.9–3.4)	0.09
Age [70–79)	2.2 (1.9–2.6)	<0.001	2.1 (1.1–4.0)	0.02
Age [80–89)	2.3 (1.9–2.8)	<0.001	1.2 (0.6–2.6)	0.5
Age [90–)	0.1 (0.1–0.3)	<0.001	0.7 (0.2–3.4)	0.7
Normal weight	-	Ref. Group	-	Ref. Group
Overweight	1.4 (1.2–1.7)	<0.001	1.0 (0.5–1.9)	0.4
Obesity	1.5 (1.3–1.8)	<0.001	1.0 (0.5–1.9)	0.9
Risky drinking	1.6 (1.2–1.7)	.001	1.3 (0.6–2.9)	0.1
Smoking	1.5 (1.2- 1.7)	<0.001	1.4 (0.6–1.8)	0.9

Ref. group = Reference group; HR = Hazard ratio; HR adjusted by gender, age, BMI, risky drinking and smoking.
